# Maternal Nodal inversely affects NODAL and STOX1 expression in the fetal placenta

**DOI:** 10.3389/fgene.2013.00170

**Published:** 2013-08-27

**Authors:** Hari Krishna Thulluru, Craig Park, Daniel Dufort, Gunilla Kleiverda, Cees Oudejans, Marie van Dijk

**Affiliations:** ^1^Department of Clinical Chemistry, Vrije Universiteit Medical CenterAmsterdam, Netherlands; ^2^Institute for Cardiovascular Research, Vrije Universiteit Medical CenterAmsterdam, Netherlands; ^3^Division of Experimental Medicine, McGill University Health CentreMontreal, QC, Canada; ^4^Department of Gynecology, FlevoziekenhuisAlmere, Netherlands

**Keywords:** pre-eclampsia, extravillous trophoblast, decidua, NODAL, STOX1

## Abstract

Nodal, a secreted signaling protein from the transforming growth factor beta (TGF-β)-super family plays a vital role during early embryonic development. Recently, it was found that maternal decidua-specific Nodal knockout mice show intrauterine growth restriction (IUGR) and preterm birth. The chromosomal location of *NODAL* is in the same linkage area as the placental (fetal) pre-eclampsia (PE) susceptibility gene *STOX1*, which is associated with the familial form of early-onset, IUGR-complicated PE. As the *STOX1* linkage was originally identified in women being born from a pre-eclamptic pregnancy as well as suffering from PE themselves, the linkage could in part be caused by *NODAL*, which is why the potential maternal–fetal interaction between *STOX1* and *NODAL* was investigated. In the PE families with the *STOX1* susceptibility allele carried by the children born from pre-eclamptic pregnancies, it was found that the pre-eclamptic mothers themselves all carried the *NODAL* H165R SNP, which causes a 50% reduced activity. Surprisingly, in decidua-specific Nodal knockout mice the fetal placenta showed up-regulation of STOX1 and NODAL expression. Conditioned media of human first trimester decidua and a human endometrial stromal cell line (T-HESC) treated with siRNAs against *NODAL* or carrying the H165R SNP were also able to induce NODAL and STOX1 expression when added to SGHPL-5 first trimester extravillous trophoblast cells. Finally, a human TGF-β/BMP signaling pathway PCR-array on decidua and the T-HESC cell line with Nodal knockdown revealed upregulation of Activin-A, which was confirmed in conditioned media by ELISA. We show that maternal decidua Nodal knockdown gives upregulation of *NODAL* and *STOX1* mRNA expression in fetal extravillous trophoblast cells, potentially via upregulation of Activin-A in the maternal decidua. As both Activin-A and Nodal have been implicated in PE, being increased in serum of pre-eclamptic women and upregulated in pre-eclamptic placentas respectively, this interaction at the maternal–fetal interface might play a substantial role in the development of PE.

## INTRODUCTION

Pre-eclampsia (PE) is a human pregnancy-associated disease, characterized by maternal hypertension and proteinuria, that occurs in about 2–8% of pregnancies. It remains the leading cause of maternal and fetal morbidity and mortality with an increase in incidence each year (WHO, 2011**)**. The symptoms of PE do not present until 20 weeks of gestation onward; however, the origin can be found in the first trimester. During normal early pregnancy, trophoblast cells invade the placental bed, modifying the spiral arteries from low-flow high-resistance to high-flow low-resistance vessels in order to reach the demands of the developing fetus. In PE patients, aberrant trophoblast differentiation, limited migration and invasion of the trophoblasts into the uterus, and poor remodeling of spiral arteries, as well as excessive apoptosis are all seen during placentation (Steegers et al., 2010).

During pregnancy, a complex interface is formed between the maternal decidua and fetal placenta, and both are involved in the secretion of and responses to essential regulatory factors that modify the maternal–fetal interface surroundings to create the conditions and anatomical structures necessary to protect the mother and the fetus (Audus et al., 2002). This process is partially driven by hormones, but it has become increasingly evident that a large number of factors like cytokines, growth factors (Lunghi et al., 2007; Knofler, 2010), and also members of the transforming growth factor beta (TGF-β) superfamily (Jones et al., 2006) are essential. The TGF-β superfamily is a large family of proteins that consists of growth and differentiation factors that are responsible for cell differentiation, proliferation, and embryo development. This family includes TGF-β, Activins, Inhibins, and Nodal, which are all excreted and subsequently are able to function in a paracrine or autocrine manner.

Recently, it was found that offspring of maternal decidua-specific Nodal knockout mice showed intrauterine growth restriction (IUGR) and spontaneous preterm birth (Park et al., 2012), due to disruption of the normal parturition cascade. The chromosomal location of *NODAL* is in the same linkage area as the placental (fetal) PE susceptibility gene *STOX1*, which is associated with the familial form of early-onset, IUGR-complicated PE (van Dijk et al., 2005). The PE susceptibility linkage locus originally identified on chromosome 10q22 by microsatellite marker analysis showed matrilineal inheritance (Oudejans et al., 2004). Investigating the individual genes on this locus for imprinting features revealed *NODAL* as one of the genes with reduced expression in the androgenetic placenta, suggestive of imprinting. As the PE linkage was originally identified in women who were both born from a complicated pregnancy (PE, pregnancy induced hypertension) and suffering from PE during their own pregnancies, the microsatellite marker linkage could in part be caused by *NODAL*, which is why this study investigated the potential maternal–fetal interaction between *STOX1* and *NODAL*.

## RESULTS

### NODAL H165R SNP IN DUTCH PRE-ECLAMPTIC MOTHERS

**FIGURE 1:**
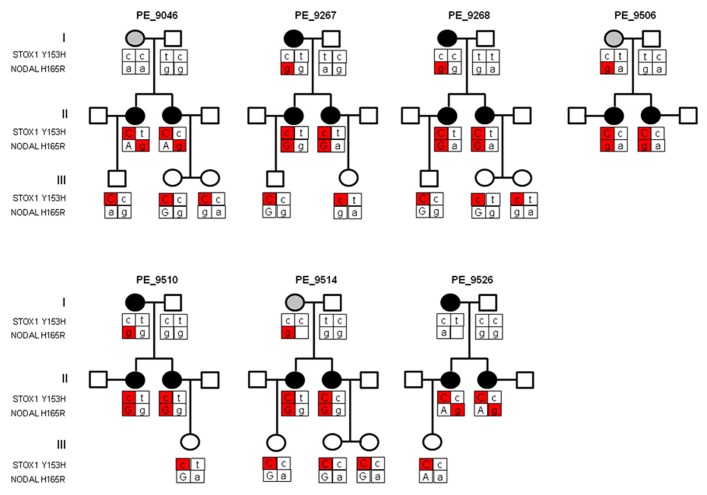
**Pedigree structure of three generations of Dutch pre-eclampsia families with linkage to chromosome 10q22.** Females in generation I had either pregnancy-induced hypertension (gray circle) or pre-eclampsia (dark circle). All sisters in generation II had pre-eclampsia and were born from pregnancies complicated by pre-eclampsia or pregnancy-induced hypertension. Generation III depicts the children born from pre-eclamptic pregnancies. Where the parental origin of the allele could be determined the maternal allele is depicted by capital letters. The *STOX1* and *NODAL* SNPs contributing to the pre-eclampsia phenotype, at placental and maternal level respectively, are indicated in red. As the pre-eclampsia linkage in this locus showed matrilineal transmission, only the *STOX1 Y153H* SNP with maternal origin is marked in red. Individuals born from complicated pregnancies (generation II and III) all carry the *STOX1 Y153H* SNP. Mothers who suffered from a complicated pregnancy (generation I and II) all carried the *NODAL H165R* SNP originating from the mother (marked in red), except in families PE_9046 and PE_9526 where the SNP in generation II originates from the father.

Previously, we identified matrilineal transmission of the *STOX1 *Y153H SNP in Dutch pre-eclamptic families (van Dijk et al., 2005). STOX1 functions in the fetal placenta as it affects trophoblast invasion (van Dijk et al., 2010b). Indeed, the *STOX1 *Y153H SNP was identified to occur in children born out of pre-eclamptic pregnancies in all families investigated. Sequencing of the Nodal gene in the same PE families used to identify the *STOX1* Y153H SNP revealed that all pre-eclamptic mothers, who gave birth to a child carrying the *STOX1 *Y153H SNP, themselves carried the *NODAL* H165R SNP (rs1904589; **Figure 1**). This indicates that the *NODAL* H165R SNP might affect the pre-eclamptic phenotype at the maternal level. *NODAL* potentially also shows a parent-of-origin effect. Further evidence was provided by the pedigrees where the allele containing the *NODAL* H165R SNP in women who suffered from a complicated pregnancy originated from their mother in five out of seven families. This SNP, although very common with a heterozygosity of around 50% in Caucasians of European ancestry, has been described to have less than 50% bioactivity (Roessler et al., 2009). In the case of a parent-of-origin effect maternal transmission of this allele would lead to a 50% reduction in activity, while normal transmission would result in about 25% less activity in heterozygotes.

### UTERINE NODAL KNOCKOUT MICE SHOW UPREGULATION OF NODAL IN THE FETAL PLACENTA

**FIGURE 2:**
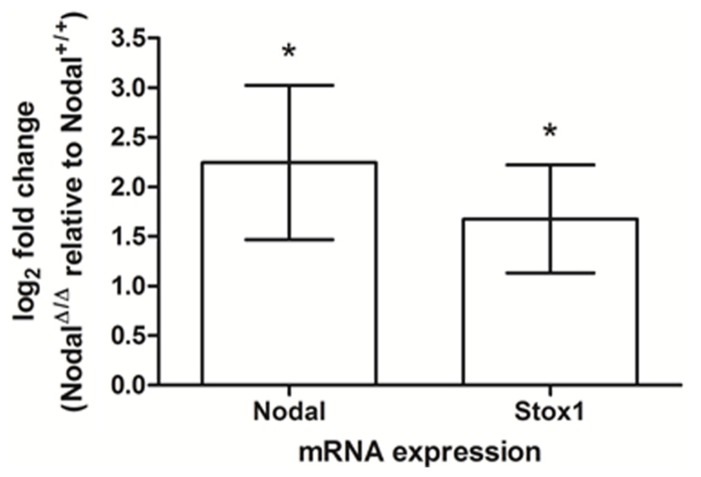
***Nodal* and *Stox1 *mRNA expression in mouse placental tissue (d16.5).** *Nodal *and* Stox1 *mRNA expression in the fetal part of the placenta of uterine Nodal knockout (*Nodal*^Δ^^/^^Δ^; *n* = 3) relative to control (*Nodal*^+^^/^^+^; *n* = 3) mice. *P < 0.05.

To gain insight into the potential interaction between *NODAL* and *STOX1* we examined the expression of *Nodal *and *Stox1* mRNA in the fetal part of the placenta of a transgenic mouse strain with maternal-specific deletion of Nodal in decidua. In short, tissue-specific conditional knockout was accomplished by utilizing a loxP flanked Nodal strain and progesterone receptor (*Pgr)*-Cre mice eliminating Nodal from the maternal reproductive tract at the onset of sexual maturity without altering the fetal layers of the placenta at the genomic level (Park et al., 2012). Interestingly, these uterine Nodal knockout mice (*Nodal*^Δ^^/^^Δ^) showed significant upregulation of *Nodal* and *Stox1 *mRNA expression**(**Figure 2**) in the fetal placenta (d16.5) compared to control mice (*Nodal*^+^^/^^+^). Maternal uterine tissues showed downregulated expression of *Nodal* upon knockout as expected (data not shown).****

### HUMAN DECIDUA SHOWS SIMILAR EFFECTS AS SEEN IN MICE

**FIGURE 3:**
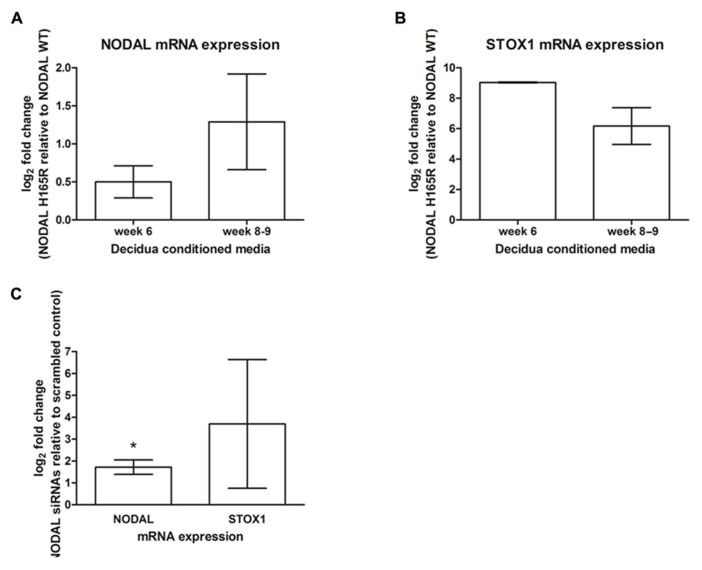
**
NODAL and STOX1 expression in SGHPL-5 cells after addition of decidual tissue conditioned media.** *NODAL* **(A)** and *STOX1* **(B)** mRNA expression in SGHPL-5 cells treated with decidua conditioned media from gestational age-matched week 6 (*n* = 4) and week 8–9 (*n* = 4) decidua tissues carrying Nodal H165R (*n* = 2 per gestational age) relative to wildtype (WT; *n* = 2 per gestational age) alleles. **(C)** *NODAL* and *STOX1* mRNA expression in SGHPL-5 cells treated with decidua conditioned media from deciduas treated with Nodal siRNAs relative to scrambled control (*n* = 4). *P < 0.05.

We tested if the effects of decidual Nodal knockdown on placental expression as found in mice could also be seen in the human placenta. For this, we collected first trimester decidual tissues carrying either wildtype or *NODAL* H165R SNP alleles. Conditioned media from these gestational age-matched decidual tissues (week 6 and week 8–9) were added to SGHPL-5 cells, a first trimester extravillous trophoblast cell line. These cells subsequently showed upregulation of both *NODAL* and *STOX1* expression when the conditioned media was from decidual tissue carrying the H165R SNP alleles (**Figures 3A,B**). In this small number of decidua tissues (*n* = 2 in each group), statistical significance was reached for *STOX1* expression showing upregulation at a gestational age of 6 weeks, while the observed upregulated *STOX1* expression at gestational age 8–9 weeks and upregulated *NODAL* expression at both gestational age timepoints did not reach statistical significance. SGHPL-5 cells incubated with conditioned media of decidual tissues (week 8–9) treated with siRNAs against Nodal or scrambled controls showed significant upregulation of *NODAL*, while the upregulated *STOX1 *expression observed did not reach statistical significance**(**Figure 3C**).

### LOCALIZATION OF NODAL IN HUMAN DECIDUA

**FIGURE 4:**
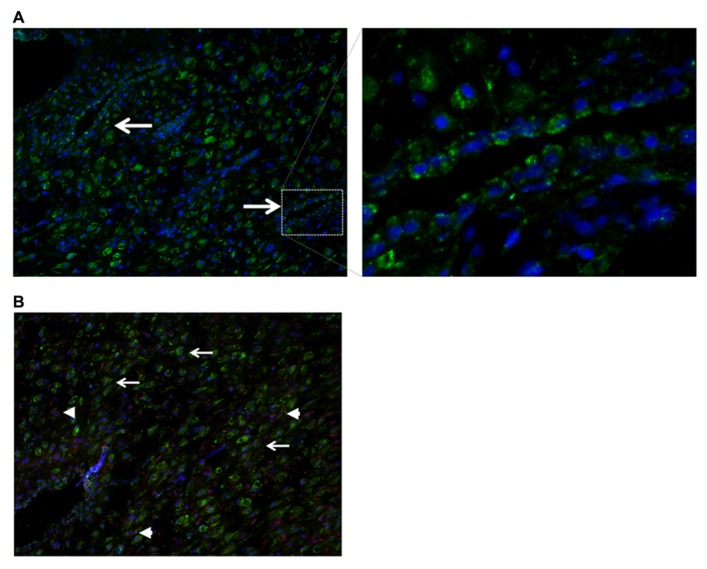
**Localization of Nodal in human first trimester decidua.** **(A)** Immunohistochemistry showing expression of Nodal (green) in epithelial glandular cells. **(B)** Localization of Nodal (green) in placental derived extravillous trophoblast cells [positive for both Nodal (green) and cytokeratin 7 (red), arrowheads] and decidual stromal cells [positive for Nodal (green), negative for cytokeratin 7 (red), arrows].

Nodal in mice was reported to be expressed in glandular epithelial cells prior to implantation, while after implantation Nodal was detectable in the outer stromal region before eventually becoming restricted to the decidua parietalis of the mature maternal decidua (Park and Dufort, 2011). Although Nodal expression has been reported to be expressed in the human placenta throughout pregnancy (Roberts et al., 2003), Nodal has not yet been investigated in human first trimester decidua. By immunohistochemistry of first trimester decidua tissues human Nodal was found to be localized in glandular epithelial cells (**Figure 4A**), placental derived extravillous trophoblast cells, and in decidual stromal cells (**Figure 4B**), similar to that detected in mice.

### UNDIFFERENTIATED DECIDUAL STROMAL CELLS CAUSE THE EFFECTS SEEN IN PLACENTA

**FIGURE 5:**
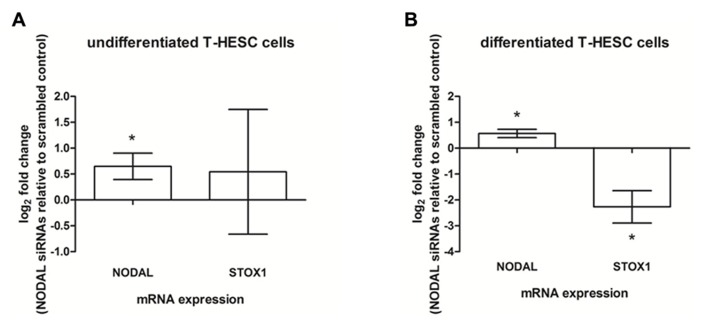
**Expression in SGHPL-5 cells after addition of conditioned media of T-HESC cells.** **(A)** *NODAL* and *STOX1* mRNA expression in SGHPL-5 cells after addition of conditioned media from undifferentiated T-HESC cells treated with siRNAs against Nodal relative to scrambled control (*n* = 5). **(B)** *NODAL* and *STOX1* mRNA expression in SGHPL-5 cells after addition of conditioned media from differentiated T-HESC cells treated with siRNAs against Nodal relative to scrambled control (*n* = 3). *P < 0.05.

As Nodal was found to be expressed in both glandular epithelial cells and decidual stromal cells, human decidual cell lines were used to see which of the two cell types were at the origin of the effects observed. Ishikawa cells, a cell line representative of glandular epithelial cells were tested by using Nodal siRNA knockdown after which the conditioned media was added to SGHPL-5 cells after which *NODAL* and *STOX1* mRNA expression levels were measured. The conditioned media of Ishikawa cells did not yield similar effects to the effects seen when conditioned media of decidual tissues were used (data not shown). A human endometrial stromal cell line, T-HESC, which can be differentiated to form decidualized cells, was used undifferentiated as a model for endometrial stromal cells and differentiated as a model for decidualized cells. In undifferentiated T-HESC cells, siRNA treatment against Nodal led to a significant upregulation of *NODAL *mRNA in SGHPL-5 cells after the addition of T-HESC conditioned media, while *STOX1* expression levels were highly variable (**Figure 5A**). Addition of conditioned media from differentiated T-HESC cells treated with Nodal siRNAs to SGHPL-5 cells were able to upregulate *NODAL*, while *STOX1 *mRNA expression was downregulated (**Figure 5B**).

### ACTIVIN-A AS SIGNALING FACTOR BETWEEN DECIDUA AND PLACENTA

We used a human TGF-β/BMP signaling pathway PCR array to investigate potential molecules responsible for upregulation of *NODAL* and *STOX1* as seen upon treatment with conditioned media of decidual tissues and cells with reduced Nodal activity or expression. Using this array we studied the mRNA expression profile of undifferentiated T-HESC cells as well as week 8 decidua tissue after transfection with siRNAs against Nodal and scrambled controls.

**FIGURE 6:**
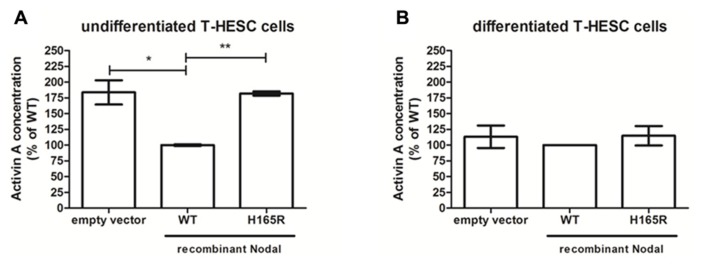
**Activin-A in conditioned media from undifferentiated and differentiated T-HESC cells.** Activin-A concentration levels in conditioned media from undifferentiated (*n* = 4) **(A)** and differentiated (*n* = 2) **(B)** T-HESC cells transfected with empty vector, wildtype (WT) or H165R Nodal constructs. *P < 0.05; **P < 0.01.

One-third (26 genes) of the genes present on the array showed differential expression between scrambled and Nodal siRNAs in both cells and tissue. Our interest was focused toward ligands as they are excreted and therefore could potentially function as signaling molecule between decidua and placenta. Nodal expression as seen in SGHPL-5 cells is initiated by ligands binding to membrane-bound Activin receptors, therefore our choice of candidate ligands was based on their ability to either activate or inhibit Activin receptors. The differentially expressed mRNAs that fulfilled these requirements were INHBA, INHBB, GFD3, and FST, which were all upregulated in the Nodal siRNA treated samples compared to scrambled controls. INHBA codes for Activin-A, and as Activin-A has been implicated in PE being increased in maternal serum of pre-eclamptic patients (Giguere et al., 2010), we decided to verify the excretion of this ligand. We performed verification of excretion by ELISA on the conditioned media of decidua tissues and T-HESC cell lines transfected with siRNAs. Following siRNA treatment, there was no measurable difference in the level of Activin-A in the conditioned media from Nodal siRNA-treated cells or decidua compared to those treated with scrambled siRNAs. Therefore, T-HESC cells transfected with wildtype or H165R Nodal constructs were used which lead to an overexpression of Nodal with full bioactivity (wildtype) or 50% reduced bioactivity (H165R). Conditioned media of H165R-Nodal in undifferentiated T-HESC cells showed significantly higher expression levels of Activin-A compared to wildtype (**Figure 6A**). Furthermore, compared to transfections with empty vector, conditioned media of wildtype Nodal transfected cells reduced the expression of Activin-A significantly. Differentiated T-HESC cells did not show any difference in expression level between empty vector, wildtype and H165R (**Figure 6B**).

## DISCUSSION

**FIGURE 7:**
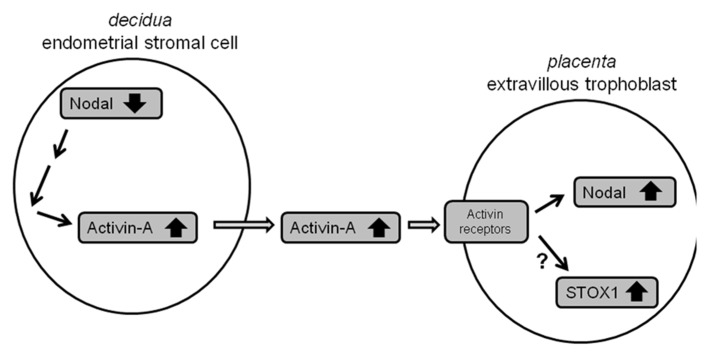
**Model of the interaction between maternal Nodal and fetal Nodal at the maternal–fetal interface.** Reduced expression of Nodal in endometrial stromal cells in the decidua, for instance due to *NODAL-H165R* alleles carried, leads to upregulation of Activin-A. Excreted Activin-A is subsequently able to upregulate Nodal, and potentially STOX1, in fetal extravillous trophoblasts.

Work performed in this study shows that maternal Nodal is able to regulate fetal placental *NODAL* and, potentially, *STOX1* expression through Activin-A excretion at the maternal–fetal interface. Firstly, we identified that pre-eclamptic mothers whose children carry the *STOX1* Y153H SNP themselves carry the *NODAL* H165R SNP, which exhibits significantly reduced bioactivity (Roessler et al., 2009). Interestingly, mice with uterine-specific knockout of Nodal in the maternal decidua have increased levels of *Nodal* and *Stox1* mRNA expression in the fetal placenta. In humans, conditioned media of first trimester decidual tissues carrying the *NODAL* H165R SNP or transfected with Nodal siRNAs were also able to upregulate *NODAL* and *STOX1* in SGHPL-5 cells, representative of first trimester extravillous trophoblast cells. This study also shows for the first time that Nodal in human first trimester decidua is localized in glandular epithelial and decidual stromal cells. As it was not known which of these two cell types was the cause of the effects seen in trophoblast cells when conditioned media of decidual tissues were used, cell lines representative of both cell types were investigated in more detail. Glandular epithelial cells were represented by Ishikawa cells, but these were found not to give the effects observed as seen when using decidual tissues. For decidual stromal cells the T-HESC cell line was used which is able to differentiate from an endometrial stromal toward a decidualized cell type, similarly as it occurs in first trimester decidual tissue during placental development. From the results obtained it can be concluded that undifferentiated T-HESC cells, representing endometrial stromal cells, cause the effects observed in decidual tissues as described above. Furthermore, it can be concluded that the upregulation of Nodal, and potentially STOX1, in the fetal placenta upon knockdown of Nodal in the maternal decidua is established, at least in part, by upregulation of Activin-A. **Figure 7** shows the model proposed on how this biological interaction at the maternal–fetal interface occurs.

Our findings nicely fit into the current knowledge regarding Nodal and Activin-A involvement in PE; in this study we provided evidence that downregulation of Nodal in decidua increases *NODAL* expression in fetal trophoblast cells. In mice decidual Nodal downregulation causes PE related symptoms of IUGR and preterm birth (Park et al., 2012), while in human pre-eclamptic mothers reduced bioactivity of Nodal is suggested as they carry the *NODAL-H165R* SNP. Several studies have suggested and shown that Nodal plays an important role during placental development (Wang and Tsang, 2007). For example, it has been shown that Nodal upregulation induces apoptosis and inhibits proliferation of a trophoblast cell line (HTR8/SVNeo) leading to reduced invasion (Munir et al., 2004). More importantly, Nodal has also been found to be increased in placentas from pre-eclamptic patients (Nadeem et al., 2011). In this study we furthermore observed that reduced Nodal activity in the maternal decidua induced Activin-A transcription leading to increased levels of Activin-A excretion. Multiple studies have found increased Activin-A concentrations in maternal serum of pre-eclamptic patients (Giguere et al., 2010). Although we did not provide direct evidence that the increased levels of Activin-A are causing the upregulated *NODAL* expression in trophoblast cells, this has recently been shown in another study where it was observed that increased Activin-A levels induce Nodal expression and its subsequent signaling in trophoblast cells (Yu et al., 2012). We are therefore confident to conclude that the Nodal upregulation in trophoblasts is, at least in part, caused by Activin-A upregulation in the decidua due to reduced Nodal activity in this tissue.

To summarize, we show in this study that maternal Nodal inversely affects fetal *NODAL* and *STOX1* expression levels, potentially through Activin-A signaling. As both Nodal and Activin-A have been implicated in PE this interaction at the maternal–fetal interface might play a substantial role in the development of PE.

## MATERIALS AND METHODS

### NODAL GENOTYPING IN PRE-ECLAMPSIA PATIENTS

Genomic DNA of the Dutch pre-eclampsia families, as described in van Dijk et al., 2005, was sequenced to identify nucleotide variations in exons of the *NODAL* gene. For sequencing, PCR fragments were purified and subjected to cycle sequencing using Big Dye terminators and analyzed using a Genetic Analyzer 3130xl (Applied Biosystems). Primer sequences are available upon request.****

### qPCR ON UTERINE-SPECIFIC NODAL KNOCKOUT MICE TISSUES

The generation of uterine specific Nodal knockout in mice has been previously described (Park et al., 2012). In short, mice with loxP sites flanking exons 2 and 3 of the *Nodal* gene (*Nodal*^lox^^P/^^loxP^) and *Pgr*-Cre mice (*Pgr*^Cre^^/^^+^) were crossed, and double heterozygote offspring (*Nodal*^loxP/^^+^, *Pgr*^Cre^^/^^+^) were crossed with *Nodal*^loxP/^^+^, *Pgr*^+^^/^^+^ mice to acquire the tissue-specific, conditional knockout strain (*Nodal*^Δ^^/^^Δ^) and controls (*Nodal*^+^^/^^+^). Uterine horn tissues of control (*Nodal*^+^^/^^+^) and knockout (*Nodal*^Δ^^/^^Δ^) mice d16.5 (*n* = 6) were fixed in formalin. Placental disks were dissected from the uterine horns, homogenized and RNA was isolated using the FFPE RNeasy kit (Qiagen). High Capacity RNA-to-cDNA kit (Applied Biosystems) was used to obtain cDNA, which was pre-amplified using preAmp mix (Applied Biosystems) followed by quantitative PCR using the taqman universal PCR mix (Applied Biosystems) in an ABI7300. Murine-specific taqman expression assays were used recognizing *Stox1* and *Nodal *(Applied Biosystems). Normalization was done using an assay for *Gapdh*.

### HUMAN FIRST TRIMESTER DECIDUA TISSUES

First trimester deciduas were obtained at the time of elective surgical terminations of pregnancy (gestational age week 6–9). Informed consent was obtained from each patient and collections were approved by the Ethical Committee of the VU University Medical Center and the Flevoziekenhuis Almere. Tissue was collected into ice-cold PBS. After extensive washing decidua parietalis was assessed for the integrity of the apical epithelial surface to confirm decidual origin. The tissues were cut into 3 mm^3^ cubes and placed in 24 well plates (three cubes per well in 800 μl phenol-red free DMEM/F12 supplemented with pen-strep and Amphotericin B) and placed overnight at 37°C, 3% O_2_, 5% CO_2_. The second day media was refreshed. After 72 h the conditioned media were collected, centrifuged to remove dead cells and stored at -80°C. In case of genotyping for *NODAL* H165R (*n* = 8), DNA of decidua was extracted from the tissues and sequenced. In case of siRNA transfection of the decidual tissues (*n* = 4), at day 2 lipofectamine RNAiMAX (Invitrogen) was used to transfect the tissues with NODAL siRNAs (Qiagen flexitube genesolution) or scrambled control. Also after 72 h conditioned media were collected and knockdown was confirmed by quantitative RT-PCR on RNA isolated from the tissues and Western Blot on protein extracted from the tissues. Knockdown was around 50%.

### IMMUNOHISTOCHEMISTRY

Human first trimester decidual tissues from 6 to 14 weeks of pregnancy were obtained as described above. Tissues were embedded in TissueTek and stored at -80°C. Using cryostat, frozen tissue blocks were sectioned (10 μm) and fixed in acetone for 10 min. After rinsing, blocking was performed for 1 h in 0.1% blocking reagent (Roche) in wash buffer containing PBS with 0.05% (v/v) Tween-20. All slide incubations were carried out in a humidified chamber at room temperature. Following blocking, sections were incubated with Nodal antibody (1:200) or cytokeratin-7 antibody (1:500; Santacruz) for 1 h, washed and secondary antibodies (1:300; anti-rabbit alexa-fluor-546 and anti-mouse alexa-fluor-488) were added for 1 h. After washing, slides were dehydrated and mounted in Vectashield containing DAPI nuclear stain (Vector Laboratories) and examined under a microscope. For negative controls the primary antibody was omitted.

### T-HESC CELL CULTURE, DIFFERENTIATION AND TRANSFECTION

T-HESC endometrial stromal cells were obtained from ATCC and grown in phenol-red free DMEM/F12 media supplemented with charcoal treated FBS, ITS+, pen-strep, and puromycin at 37°C, 5% CO_2_. Differentiation was performed by the addition of 0.5 mM 8-Bromo-cAMP, 1 μM progesterone and 10 nM Estradiol (all obtained from Sigma) on every third day. Differentiation was complete after 12 days as observed by a change in cell morphology and a 20-fold increase in prolactin excretion as measured in conditioned media.

T-HESC siRNA transfections in undifferentiated (five independent experiments) and differentiated (three independent experiments) cells were done as described for tissue transfections. After 72 h conditioned media were collected, centrifuged to remove dead cells and stored at -80°C. Knockdown was assessed by quantitative RT-PCR and Western blotting. Knockdown of Nodal mRNA and protein was at least 75%. 

A vector to express Nodal protein was constructed in a pF5K-cmv-neo flexi vector (Promega) according to the flexi-vector protocol. The H165R SNP was introduced by using the Quickchange XL site-directed mutagenesis kit (Stratagene). Integrity of the constructs was verified by sequencing. Transfection in undifferentiated (four independent experiments) and differentiated (two independent experiments) of the constructs and empty vector was done using Fugene HD transfection reagent (Roche). After 72 h conditioned media were collected, centrifuged to remove dead cells, and stored at –80°C. Overexpression of Nodal was confirmed by quantitative RT-PCR and Western blotting.

### SGHPL-5 CELL CULTURE AND QUANTITATIVE RT-PCR

Different concentrations of conditioned media obtained from (transfected) decidual tissues and T-HESC cells were added in duplicate to 4,00,000 SGHPL-5 cells, kindly provided by Dr Judith Cartwright, St George’s University of London, UK, per well in 12 well plates. After 30 h SGHPL-5 cells were collected and RNA isolated using the RNeasy kit (Qiagen). Quantitative RT-PCRs were performed in triplicate on an ABI7300 using the Taqman EZ RT-PCR kit (Applied Biosystems) with a gene expression assay for *NODAL* (Applied Biosystems) and primers and probe recognizing STOX1 isoform A as described before (van Dijk et al., 2010a). Normalization was done with gene expression assays for *GAPDH*.

### TFG-β BMP SIGNALING PATHWAY PCR ARRAY AND ACTIVIN-A ELISA

RNA from siRNA transfected undifferentiated T-HESC cells and decidua tissue as described above was used on a TGF-β/BMP signaling pathway PCR array (SABbiosciences) according to the manufacturer’s instructions.

To confirm the results obtained by this array, Activin-A protein secretion was analyzed in duplicate in the conditioned media of transfected decidua tissues and T-HESC cells, obtained as described above, using an ELISA for Activin A (Activin-A Human ELISA kit, Abcam) according to the manufacturer’s protocol and analyzed on a Biotek synergy HT micro plate reader at 450 nm using Gen5 software.

### STATISTICS

All data are calculated and expressed as mean ± SEM. Results were subjected to statistical analysis by Student’s *t*-tests as appropriate using Prism 5.0c (GraphPad Prism, San Diego, CA, USA) and significance was accepted when *P* < 0.05. All graphs are based on the combined data of independent samples or transfections used, except the graph of 6A that shows a representative graph of one of the four independent transfections performed.

## Conflict of Interest Statement

The authors declare that the research was conducted in the absence of any commercial or financial relationships that could be construed as a potential conflict of interest.
